# Individual Differences Facing the COVID-19 Pandemic: The Role of Age, Gender, Personality, and Positive Psychology

**DOI:** 10.3389/fpsyg.2021.644286

**Published:** 2021-03-19

**Authors:** Gloria Bernabe-Valero, David Melero-Fuentes, Irani I. De Lima Argimon, Maria Gerbino

**Affiliations:** ^1^Mind, Emotion and Behavior Laboratory (MEB Lab), Faculty of Psychology, Catholic University of Valencia Saint Vincent Martyr, Valencia, Spain; ^2^Information Science and Technology Group, Faculty of Psychology, Catholic University of Valencia Saint Vincent Martyr, Valencia, Spain; ^3^Postgraduate Program in Psychology, Pontifícia Universidade Católica do Rio Grande do Sul, Porto Alegre, Brazil; ^4^Department of Psychology, Sapienza University of Rome, Rome, Italy

**Keywords:** crowdsourcing, SARS-CoV-2, positive psychology, personality, profiles, gratitude, religiosity, purpose of life

## Abstract

Research on individual differences in facing the COVID-19 pandemic seems to be crucial in order to design diverse and highly effective intervention strategies. This study uses a sample of 302 North American participants who were recruited through the crowdsourcing platform ProA; different profiles were established, profiling variables of interest in facing the COVID-19 outbreak. Socio-demographic and psychological (personality traits, gratitude, life purpose, and religiosity) variables were explored. These results are of interest if we want to deepen the study of individual differences at both a theoretical and applied level.

## Introduction

The COVID-19 pandemic was the biggest social and health crisis to occur in 2020. The scientific community is working to cure the disease, to mitigate the side effects, and to provide preventive measures, such as isolation, to reduce infection rates. Many researchers have identified the effects of the pandemic on mental health and welfare issues among the general population (Ammar et al., 2020; Zheng et al., 2020). In fact, a large study involving 35 research organizations from Europe, North-Africa, Western Asia, and the Americas, with 1,047 participants, revealed the presence of psychosocial strain and lower life satisfaction during the enforced COVID-19 lockdowns, due to the large decreases in entertainment and in the amount of social activity with family and friends/neighbors (Ammar et al., 2020). Furthermore, a national public survey in Ireland showed that COVID-19-related quarantine was associated with significant increases in clinically significant symptoms of depression, stress, and anxiety (Burke et al., 2020).

Nevertheless, a large cross-sectional survey of more than 50,000 people in 26 countries found that not everyone was equally affected by the COVID-19 pandemic (Kowal et al., 2020). The results of this study were that younger people, women, those with lower levels of formal education, those who were single, those living with more children, or living in a country more severely affected by COVID-19, exhibited higher levels of stress. Therefore, research on individual differences due to the impact of the COVID-19 pandemic is necessary in the field of psychology.

In this sense, a point of interest for the scientific community is being able to identify different profiles and how people with these profiles deal with consequences of the COVID-19 lockdown based on various personality traits and strengths relating to positive psychology. Other authors have gone further with this, investigating the factors, traits, and strengths related to well-being in the time of a pandemic. For example, the effects of lockdown and the pandemic on the general population have been explored based on age and gender. Regarding age, the general consensus is that older people exhibit a feeling of greater well-being and less negative affectivity. In this way, a study found older people rated their quality of life, life satisfaction, and well-being during the pandemic higher than young people, and experienced lower levels of anxiety traits and coronavirus fears than the younger age groups. They experienced greater risk tolerance, sleep quality, and optimism, and had less difficulty relaxing than middle-aged respondents (Bidzan-Bluma et al., 2020). Another research study has shown that age was significantly and negatively associated with initial negative affect, but age did not influence the shape or rate of change over time. Moreover, although older adults showed higher positive affect and lower negative affect relative to younger adults, age differences in the trajectory of change did not emerge (Ebert et al., 2020). In relation to age, another study carried out with a sample majority of students, between 18 and 40 years old, showed mild to severe General Anxiety Disorder, and a high level of perceived stress, however, it is not known what the reasons might be for this age-determined difference (Rogowska et al., 2020). As for gender, some research suggests that female participants may experience less satisfaction with life and higher stress and anxiety throughout the coronavirus pandemic (Wang et al., 2020). Another study indicates that the variance of anxiety during the COVID-19 outbreak may be explained up to about 60% by variables like high stress, low general self-rated health, female gender, and frequent use of both emotion-oriented and task-oriented coping styles (Rogowska et al., 2020). However, other studies conducted in China during the COVID-19 pandemic outbreak did not find gender differences in mental health. Gender had no significant effect on anxiety among medical college students (Cao et al., 2020), as well as among the general population (Huang and Zhao, 2020). Furthermore, Zhang and Ma (2020) did not find gender differences regarding the stressful impact of the COVID-19 outbreak. These research inconsistencies may be related to cross-cultural differences, so further exploration is needed in different cultural contexts.

Previous research suggests that psychological consequences of the pandemic depend on personality, because this predicts behavioral responses and emotional regulation strategies to cope with the COVID-19 crisis, and these can influence physical and psychological health (Aschwanden et al., 2020; Gubler et al., 2020). In fact, a study on individual differences in the psychological consequences of COVID-19 found that facets of Extraversion, Neuroticism, and Openness were among the strongest and most important predictors of psychological outcomes, even after controlling for basic sociodemographic variables such as gender and age (Modersitzki et al., 2020). Similarly, a study carried out during the pandemic determined that neuroticism and emotion regulation strategies were associated with greater feelings of loneliness and lower well-being (Gubler et al., 2020). Moreover, it was found that higher levels of neuroticism were associated with a slower increase in physical activity, whereas higher conscientiousness and agreeableness were related to a steeper increase in physical activity over time. As well as this, higher neuroticism and lower extraversion, agreeableness, and conscientiousness were related to higher average sedentary behavior (Aschwanden et al., 2020). In this way, it is key to highlight the importance of considering individual differences in relation to this topic.

Additionally, the study of Positive Psychology has been of great importance because positive resources can help maintain and improve mental health during the COVID-19 pandemic. This field of research has begun to show that a large percentage of the general population is capable of maintaining healthy levels of subjective and psychological well-being despite adverse circumstances, identifying the human strengths that make it possible to deal positively with adversity. For example, previous research indicates that resilient people report that one of the emotions that most effectively buffer the negative effects of adversity is gratitude (Fredrickson et al., 2003). Another testimony is that of the Dalai Lama, who was grateful to the Japanese for the harm they had inflicted on him, as it helped him develop as a person and grow spiritually (Fitzgerald, 1998). Survivors of Hurricane Andrew (1992) also reported that one of the central themes in their experience was an overwhelming sense of gratitude for what they had not lost during the hurricane (Coffman, 1996). In the wake of the tragedy of the 9/11 terrorist attacks, Peterson and Seligman (2003) assessed people before and after the event, showing that gratitude increased during this period. In addition, psychological interventions to increase gratitude had beneficial effects for Vietnam War veterans with Post-Traumatic Stress Disorder (Kashdan et al., 2006). These results suggest that gratitude may play an important role in what has recently been termed “post-traumatic growth,” referring to the benefits that can be experienced from overcoming trauma, despite the intense suffering from which it has originated (Bono et al., 2004). Specifically, several studies suggest that gratitude has an important role in promoting people's subjective well-being and helps them cope better with adversity during the COVID-19 pandemic (Bono et al., 2020a). Additionally, meaning in life has been one of the strengths associated with resilience in the face of adversity. For example, a study indicated that meaningful living had a positive predictive effect on resilience and positive affect, as well as a negative predictive value on psychological health challenges and negative affect on the psychological health of young adults in the context of the pandemic (Arslan et al., 2020). Martínez-Martí et al. (2020) evaluated the trait strengths of the general Spanish population at two points throughout the pandemic, noting that all character strength factors at point #1 correlated positively with life satisfaction and positive affect, and negatively with negative affect and poor mental health at point #2. In the health context of the pandemic, a nurse's sample (Sun et al., 2020) found self-coping styles included altruistic acts, team support, rational cognition, increased affection and gratefulness, development of professional responsibility, and self-reflection. In addition to this, Nowicki et al. (2020), also in a nurse's sample, noted an increase in life meaning, so their current sense of meaning in life remains higher than the tendency to search for a sense of security, being able to adapt to painful experiences and generate post-traumatic growth effect. On the other hand, one of the variables that has been researched by Positive Psychology and has been revealed as a buffer against crises is religiosity. For example, research has shown that throughout the pandemic, Google searches for “prayer” (relative to all Google searches), were at the highest level ever recorded, and more than half of the world's population had prayed to end coronavirus (Bentzen, 2020). Another study carried out during the pandemic found that religiosity had a positive influence on health outcomes and could minimize the effects of social isolation (Lucchetti et al., 2020). However, other studies have shown that highly religious participants reported more unreasonable behavior (e.g., avoiding 5G networks, hoarding toilet paper) than participants with low religiosity, although these behaviors were mediated through emotionality (Kranz et al., 2020). For this reason, from the Positive Psychology approach, we are specifically interested in the personal strengths of gratitude, meaning in life, and religiosity as important variables to explore in relation to coping with COVID-19.

Thus, according to the scientific knowledge that has been uncovered, and its gaps, this study seeks to provide more evidence in the field of individual differences surrounding the situation of the COVID-19 pandemic. The aim of this study is to explore the factors of age, gender, personality, and variables of Positive Psychology (gratitude, meaning or purpose in life, and religiosity) in relation to affectation in the face of the COVID-19 pandemic.

## Materials and Methods

### Design and Sample

A cross-sectional design was used. The study consisted of 302 US residents whose primary language was English. They were recruited from the Prolific ProA Platform (www.prolific.co), of which 153 (51%) were women and 149 (49%) were men. Ages ranged between 19 and 82 years old (M = 45.07, SD = 15.94).

Table 1 shows sociodemographic data such as generational breakdown and ethnicity and Table 2 shows employment status, educational level, and marital status.

**Table 1:** Sociodemographic characteristics: generational breakdown and ethnicity.

**Generational breakdown**	**Percent**	**Ethnicity**	**Percent**
18–29 years old	22	Asian	8
30–39 years old	17	Black	15
40–49 years old	15	Mixed race	5
50–59 years old	21	White	69
60 and more years old	25	Other	3

**Table 2:** Sociodemographic characteristics: employment status, educational level, and marital status.

**Employment status**	**Percent**	**Educational level**	**Percent**	**Marital status**	**Percent**
A homemaker	4.30	Bachelor's degree	42.38	Separated	4.30
A student	7.67	Doctorate degree	2.32	Single, never married	11.92
Other	1.99	Master's degree	14.24	Widowed	13.91
Out of work and looking for work	12.58	No schooling completed	16.56	Married or domestic partnership	47.02
Out of work but not currently looking for work	3.31	Professional degree	2.65	Divorced	12.25
Retired	14.90	Trade/technical/vocational training	21.85		
Salaried	34.11				
Self-employed	17.22				
Unable to work	3.97				

### Instruments

A socio-demographic survey was created for this study (employment status, educational level, marital status, age, and gender).

The English adaptation of the Gratitude Questionnaire (G20, Bernabé-Valero et al., 2014) was used in the present study (Bernabe-Valero et al., 2020). This scale has four subscales and 20 items that were rated on a 7-pt scale (“1 = Strongly Disagree” to “7 = Strongly Agree”). The scores are obtained by adding the direct scores; the range of the total scale is from 20 to 140. The first subscale is Interpersonal Gratitude (IG)—gratitude that is experienced toward other people when receiving a gift or an act of kindness. It refers to benefactors with different types of relationships to the beneficiary and focuses on the evaluative, emotional, and behavioral elements of gratitude. This subscale has seven items (e.g., “I feel great joy when someone does me an important favor”) and showed good reliability (α = 0.88). The range of scores on this subscale is 7–49. The second subscale is Gratitude in the face of Suffering (GS)—this factor refers to the integration of suffering in the concept of gratitude. It assesses the ability to understand situations of suffering as beneficial and to feel gratitude nonetheless. Likewise, it assesses if the person is able to move forward despite difficulties and to use gratitude as a resource for resiliency. It includes the cognitive-evaluative and emotional elements of gratitude. This subscale has five items (e.g., “Even after times in my life when I only experienced suffering, I can feel gratitude for having had the strength to get through them”) and showed excellent reliability (α = 0.92). The range of scores on this subscale is 5–35. The third subscale is Recognition of Gifts (RG)—awareness of the positive aspects of existence while considering them as gifts and implicitly attributing these gifts to a transpersonal agent (e.g., destiny, luck, nature, or divine providence). It includes the process that leads to the recognition of assets and their appraisement, as well as the social comparison that gives rise to the awareness of the positive aspects in one's life. This subscale has four items (e.g., “Every day I am aware that the little things in life that happen to me are a gift”) and showed good reliability (α = 0.87). The range of scores on this subscale is 4–28. The fourth subscale is Expression of Gratitude (EG): the experience and expression of gratitude toward transpersonal forces. Forms of expression can be verbal expression, rituals, and an attitude toward life of trying to be happy. This subscale has four items (e.g., “When I ask God or Fortune for help and I receive it, I usually remember those favors and give thanks”) and showed acceptable reliability (α = 0.79). The range of scores on this subscale is 4–28. The G20 obtained good reliability indices in its construction with a Spanish sample. Cronbach's alpha for each subscale was good and acceptable (IG α = 0.84, GS α = 0.78, RG α = 0.75, EG α = 0.75).

Ten-Item Personality Inventory (TIPI) (Gosling et al., 2003) is a 10-item measure of the Big-Five dimensions. Each item consists of two descriptors, separated by a comma, using the common stem, “I see myself as (i.e., extraverted, enthusiastic)”. Each of the 10 items was rated on a 7-point scale ranging from 1 (disagree strongly) to 7 (agree strongly). The TIPI takes about a minute to complete. The score is obtained from the sum of the two items for each subscale after reversing an item in each of them. The range of scores for each subscale is 2–14. The Cronbach alphas in the original version were 0.68, 0.40, 0.50, 0.73, and 0.45 for the Extraversion (E), Agreeableness (A), Conscientiousness (C), Emotional Stability (ES), and Openness to Experience (OE) scales, respectively. Alpha Cronbach in this sample was 0.73, 0.43, 0.77, 0.81, and 0.46, respectively.

The Revised Purpose in Life questionnaire (PIL-R; Harlow et al., 1987) was revised from one developed by Crumbaugh (1968), based on Frankl's (1985) existential perspective. This work used the bifactorial version (García-Alandete et al., 2011) with two scales: Satisfaction and Sense of Life (SSL) with six items, four of which are reversed (i.e., “I am usually completely bored”), and Goals and Purposes in Life (GPL) with four items, two of which are reversed (i.e., “In life I have no goals or aims at all”). The bifactorial structure of García-Alandete et al. (2011) obtained good internal consistency, both for the scale (α = 0.86) and for the factors (α = 0.84 and α = 0.71), respectively. Alpha Cronbach in this sample was 0.81 and 0.75, respectively. The PIL-R assesses the degree to which an individual has a sense of meaning or purpose in life. 7-point Likert scales ranging from “strongly disagree” (1) to “strongly agree” (7) are used to answer the items. Items refer to having goals or aims, life being empty or worthwhile, a sense of boredom or excitement, free will, despair, and sense of satisfaction with life. The score is obtained from the sum of the items for each subscale after reversing the items in each of them. The range of scores for each subscale is 6–42 (SSL) and 4–28 (GPL).

Brief Scale of Religiosity (BSR) (Bernabé-Valero et al., 2015) is a one-dimensional scale, comprising four items. It is a self-administered scale. Religious self-definition, degree of personal religiosity, frequency of attendance at worship and prayer, and importance of God in one's life are measured (i.e., “In terms of religion, I consider myself…”: “1-Not religious at all” to “6-Extremely Religious”). No specific religious denomination is specified in the items. The response options are ordered from lesser to greater degree of religiousness with six levels of response (i.e., from “1-Not religious” to “6-Extremely religious”). The score is obtained from the sum of the four items and the scores range from 6 to 36. The Cronbach alphas in the original version were good (α = 0.89) and very similar in this sample (α = 0.90).

#### COVID-19's Impact Was Assessed Using the Following Two Instruments

The affective experience in the pandemic situation was assessed using the Positive Affects and Negative Affects Schedule (PANAS) (Watson and Clark, 1999). It's a comprehensive mood inventory originating from a dimensional approach to the understanding of emotion. It includes 10 items for Negative Affect (NA) and 10 items for Positive Affect (PA). Items are measured on a 5-point Likert scale, from “1-very slightly or not at all” to “5-extremely.” In order to measure mood during the pandemic, we used the version PANAS-X (Past few weeks), in which the participants were asked to indicate to what extent they have felt this way over the past few weeks. The NA subscale comprises the following items: Distressed, Irritable, Jittery, Ashamed, Hostile, Guilty, Upset, Scared, Nervous, and Afraid. The PA subscale comprises the following items: Active, Enthusiastic, Determined, Attentive, Inspired, Strong, Interested, Alert, Excited, and Proud. Internal Consistency Reliabilities (Coefficient Alpha) in its construction were PA (α = 0.87) and NA (0.87) for undergraduates, and PA (α = 0.86) and NA (0.87) for employees. In the present study, the reliability is (α = 0.90) in PA and (α = 0.91) in NA. The score is obtained from the sum of the items for each subscale. The range of scores for each subscale is 5–50.

Also, COVID-19 affectation was measured with the question: “We are currently in a worldwide pandemic situation due to COVID-19. Has this significantly affected your mood and emotions?” The three possible answers were: “Yes, I am feeling worse,” “No, no change or almost no change,” “Yes, I am better.” We label this variable as “Facing COVID” (FC).

### Statistical Analysis

Several tests were carried out in order to determine the relationships. Pearson's chi-squared test (χ^2^) was carried out with dichotomous (gender) and ordinal variable (FC), Spearman's Rank Correlation Coefficient (rho) when the variable involved was FC (ordinal), and Pearson correlation coefficient (r) with metric variables. Student's *T*-test was used to explore the differences between men and women with metric variables.

With the aim of identifying whether there are different participant profiles with affectation of COVID-19 and target variables, several two-step cluster analyses were carried out with affectation of COVID-19 (FC and PANAS variables) and (i) age, (ii) gender, (iii) personality traits, (iv) positive and negative effects, (v) gratitude subscales, (vi) purpose in life subscales, and (vii) religiosity.

Since the cluster selection procedure is analytical, we proceeded to identify the quality of the clusters, mainly by looking at the “cluster quality” and “predictor importance” indicator in order to choose those that are useful for the research objectives. According to Kaufman and Rousseeuw (1990), a result in the fair zone means that the data give fair evidence of this cluster structure. The second most relevant indicator is the importance of the predictors, since, even if a cluster is good or fair, if there is only one predictor variable with high importance, the model is not useful, since the objective is to identify profiles with several variables. Therefore, clusters that meet at least two criteria are selected: (a) the criterion that the model has a fair or good outcome and (b) that at least two predictors have an importance >0.04. In this sense, only three cluster analyses are accepted to continue with analysis: the cluster that includes the variables referring to COVID affectation together with gender, the cluster that includes the variables referring to COVID affectation and gratitude, and the cluster that includes the variables referring to COVID affectation and purpose in life.

Since the Kolmogorov-Smirnov test highlighted that the ordinal and metric variables did not have a normal distribution, the tests used to check the quality of the clusters selected were as follows: (i) Kruskal–Wallis *H*-test and Mann-Whitney *U*-test with Bonferroni correction (alfa = 0.005 in cluster #1 and alfa = 0.017 in clusters #4 and #5) and (ii) χ^2^-test with variable gender (dichotomous) in cluster #1.

## Results

FC is not associated with gender χ${}_{\left(2\right)}^{2}$ = 0.210, *p* = 0.901 (Table 3) and age rho = −0.068, *p* = 0.238. Table 3 shows that 51.66% (*n* = 156) was marked “worse” in FC followed by: no change” 41.72% (*n* = 126) and “better” 6.62% (*n* = 20). There are no value differences between men and women for FC. There are also no differences in gender with respect to affectivity: in PA (positive affects), *F*_(300)_ = 0.148, *p* = 0.294. For NA (negative affects), *F*_(300)_ = 0.333, *p* = 0.316.

**Table 3:** FC and gender co-occurrences.

	**Better *n* (%)**	**No change *n* (%)**	**Worse *n* (%)**	**∑*n* (%)**
Man	10 (3.31)	64 (21.19)	75 (24.83)	149 (49.34)
Woman	10 (3.31)	62 (20.53)	81 (26.82)	153 (50.66)
∑	20 (6.62)	126 (41.72)	156 (51.66)	302 (100)

FC is inversely correlated with all personality traits among −0.003 (EX) and −0.339 (ES) (see all rho values in Table 4). Only the correlations with ES and CO are significant.

**Table 4:** Spearman's Rank correlation coefficient between FC and personality variables.

	**EX extraversion**	**AG agreeableness**	**CO conscientiousness**	**ES emotional stability**	**OE openess to experiences**
Rho	−0.003	−0.086	−0.132	−0.339	−0.080
*p*-value	0.960	0.134	0.021	0.000	0.166

There is no signification correlation between FC and PA and NA (Table 5). Positive Psychology variables (gratitude, purpose in life, and religiosity) also failed to obtain significant correlations with FC. The rho values are between 0.062 and −0.060 (see all rho values in Table 6).

**Table 5:** Pearson correlation coefficient between FC and positive and negative affects.

	**PA positive affects**	**NA negative affects**
Rho	−0.095	−0.042
*p*-value	0.098	0.465

**Table 6:** Spearman's Rank Correlation Coefficient between FC and Positive Psychology variables.

	**BSR religiosity**	**IG interpersonal gratitude**	**GS gratitude in the face of suffering**	**RG recognition of gifts**	**EG expression of gratitude scale**	**SSL satisfaction and sense of life**	**GPL goals and purposes in life**
Rho	−0.058	0.033	−0.035	−0.042	−0.060	0.062	0.030
*p*-value	0.318	0.563	0.545	0.468	0.301	0.191	0.538

Table 7 shows the associations for all the metric variables explored. The affective lived experience during the pandemic measured through the PANAS correlated significantly with most of the Positive Psychology variables (BSR, GI, GS, RG, EG, SSL, and GPL). Positive Affects (PA) were positively and significantly associated with the four subscales of Gratitude (IG, GS, RG, and EG), Satisfaction and Sense of Life (SSL), Goals and Purposes in Life (GPL), and Religiosity. PA had no significant associations with age and personality traits. NA obtained significant negative associations with the four subscales of Gratitude (IG, GS, RG, and EG), Satisfaction and Sense of Life (SSL), Goals and Purposes in Life (GPL), and Religiosity. It also has significant negative associations with age. There are not many high correlations between personality traits and the PANAS. Positive Affect (PA) was not associated with any personality trait; Negative Affect (NA) was only significantly and negatively associated with emotional stability. Pearson scores among Positive Psychology variables are between −0.197 and 0.753, where there are only two notable correlations: Gratitude Expression and Religiosity (0.720) and Recognition of Gifts and GS (0.753) followed below by four correlations scoring between 0.5 and 0.6.

**Table 7:** Pearson correlation coefficient between age, personality traits, Positive and Negative affects, and positive psychology variables.

		**IG**	**GS**	**RG**	**EG**	**SSL**	**GPL**	**BSR**	**PA**	**NA**	**Age**	**E**	**A**	**C**	**ES**
GS	*r*	0.473													
	*p*	0.000													
RG	*r*	0.584	0.753												
	*p*	0.000	0.000												
EG	*r*	0.351	0.539	0.594											
	*p*	0.000	0.000	0.000											
SSL	*r*	−0.162	−0.085	−0.204	−0.073										
	*p*	0.005	0.142	0.000	0.207										
GPL	*r*	0.007	−0.017	−0.056	0.016	0.331									
	*p*	0.906	0.771	0.329	0.783	0.000									
BSR	*r*	0.209	0.336	0.335	0.720	−0.039	−0.013								
	*p*	0.000	0.000	0.000	0.000	0.498	0.825								
PA	*r*	0.268	0.496	0.519	0.404	−0.165	−0.050	0.229							
	*p*	0.000	0.000	0.000	0.000	0.004	0.385	0.000							
NA	*r*	−0.200	−0.316	−0.371	−0.285	0.372	0.120	−0.147	−0.456						
	*p*	0.000	0.000	0.000	0.000	0.000	0.037	0.010	0.000						
Age	*r*	0.078	0.021	0.189	0.225	−0.287	−0.113	0.236	0.047	−0.266					
	*p*	0.179	0.721	0.001	0.000	0.000	0.052	0.000	0.420	0.000					
E	*r*	−0.036	0.031	−0.039	−0.037	−0.103	0.053	−0.044	0.033	−0.072	0.054				
	*p*	0.533	0.597	0.496	0.516	0.075	0.358	0.445	0.573	0.213	0.349				
A	*r*	−0.010	−0.048	−0.018	0.007	0.003	−0.087	0.025	−0.088	−0.026	0.202	0.170			
	*p*	0.867	0.407	0.755	0.910	0.957	0.132	0.661	0.127	0.657	0.000	0.003			
C	*r*	−0.023	−0.050	−0.041	0.014	−0.150	0.007	0.055	0.058	−0.110	0.180	0.205	0.285		
	*p*	0.692	0.388	0.476	0.809	0.009	0.907	0.343	0.311	0.056	0.002	0.000	0.000		
ES	*r*	0.051	−0.004	0.025	0.018	−0.197	−0.058	0.018	0.085	−0.158	0.182	0.333	0.375	0.538	
	*p*	0.381	0.950	0.663	0.759	0.001	0.313	0.753	0.138	0.006	0.002	0.000	0.000	0.000	
OE	*r*	−0.007	−0.031	−0.010	0.042	−0.063	−0.094	−0.047	0.031	−0.049	0.118	0.363	0.313	0.196	0.303
	*p*	0.906	0.597	0.858	0.472	0.276	0.104	0.419	0.588	0.392	0.041	0.000	0.000	0.001	0.000

Table 8 shows the main indicators for each cluster analysis performed. More information is available in Appendix 1.

**Table 8:** Clusters FC and positive and negative affects with age, gender, personality traits, gratitude, purpose in life, and religiosity.

**Set of variables included in the clusters two-steps analysis (inputs)**	**Cluster quality (poor. fair. good)**	**Average silhouette measure of cohesion and separation**	**Number of clusters**	**Ratio of sizes of clusters**	**Feature importance**	**Utility decision**
FC+ PA+ NA+ gender	Fair	0.5	5	4.05	1; 0.47; 0.01; 0.00	selected
FC+ PA+ NA+ age	Fair	0.3	2	1.02	1; 0.01; 0.01; 0.01	rejected
FC+ PA+ NA+ Personality traits (E+A+C+ES+OE)	Fair	0.3	2	1.40	1; 0.12; 0.03; 0.01; 0.0	rejected
FC+ PA+ NA+ Gratitude (IG+GS+RG+EG)	Fair	0.3	3	2.58	1; 0.92; 0.58; 0.53; 0.23; 0.27; 0.11	selected
FC+ PA+ NA+ +Purpose in Life (SSL+GPL)	Fair	0.3	2	1.40	1; 0.01; 0.01; 0.0; 0.0	selected
FC+ PA+ NA+ Religiosity (BSR)	Fair	0.3	2	1.07	1; 0.01; 0.01; 0.01	rejected

The first selected cluster analysis included the variables of COVID affectation and gender. The five obtained clusters are described below:

Cluster #1 comprises 26.8% of the participants (*N* = 81), 100% belong to the category “worse” in their COVID-19 affect, and 100% are women with medium-high scores in positive affect (mean = 43.26) and medium-low scores in negative affect (mean = 21.22). They are “women worse in COVID medium affect.”

Cluster #2 comprises 6.6% of the participants (*n* = 20), 100% belong to the category “better” in their COVID-19 affect, 50% are female and 50% are male, and their scores are high in positive affect (mean = 46.15) and medium-low in negative affect (mean = 18.10). They are labeled as “best mixed gender group in COVID-19 good affect.”

Cluster #3 comprises 20.5% of the participants (*N* = 62), 100% belong to the category “no change” in their COVID-19 affect, and 100% are women with high scores in positive affect (mean = 46.71) and low scores in negative affect (mean = 19.44). They are labeled as “women with no change in COVID good affect.”

Cluster #4 comprises 21.2% of the participants (*N* = 64), 100% belong to the category “no change” in their COVID-19 affect, and 100% are men with high scores on positive affect (mean = 47.19) and low scores on negative affect (mean = 19.44). They are labeled as “men with no change in COVID affect.”

Cluster #5 comprises 24.8% of the participants (*N* = 75), 100% belong to the category “worse” in their COVID-19 affect, and 100% are men with medium-high scores on positive affect (mean = 45.92) and low scores on negative affect (mean = 18.69). They are labeled as “men worse in COVID medium-positive affect.”

Kruskal-Wallis *H*-test indicates significant differences for FC (*p* < 0.001) in the first selected cluster analysis, while PA *p* = 0.141 and NA *p* = 0.346. Mann Whitney *U*-test in FC indicates that all differences between clusters are significant (*U* < 0.001, *p* < 0.001) except between clusters #1 and #5 (*U* = 3,034, *p* = 1.000) and #3 and #4 (*U* = 1,984, *p* = 1.000). χ^2^-test for gender and first cluster analysis shows the relationship between these variables [χ${}_{\left(4\right)}^{2}$ = 278.045, *p* < 0.001].

The second selected cluster analysis included the variables of COVID affectation and gratitude variables. The three obtained clusters are described below:

Cluster #1 (*n* = 50, 16.6%) comprises 54% of participants who report feeling worse since the pandemic started, the remaining 46% are distributed between no change (32%) and better (14%). In gratitude, they obtained medium scores in RG (mean = 16.32), medium-high scores in IG (mean = 36.22), and low scores in GS (mean = 16.56) and EG (mean = 10.68). In relation to affect, they obtain medium-low scores in positive affect (mean = 35.08) and medium-high scores in negative affect (mean = 25.24). We will label it as “mixed on medium gratitude and medium affect.”

Cluster #2 (*n* = 123, 40.7%) comprises 89.4% of participants reporting no change since the pandemic started and the remaining 10.6% are better. In gratitude they score high on RG (mean = 25.08) and IG (mean = 43.91), medium-high on GS (mean = 28.14), and medium on EG (mean = 18.91). In relation to affect, they scored high in positive affect (mean = 48.51) and low in negative affect (mean = 18.91). We will label it as “no COVID affect, medium-high gratitude, and good affect.”

Cluster #3 (*n* = 129, 42.7%) comprises 100% of participants who report being worse since the pandemic started. In gratitude they scored high on RG (mean = 24.85) and IG (mean = 44.32) and medium-high on GS (mean = 28.12) and medium on EG (17.95). In relation to affect, they scored high in positive affect (mean = 46.54) and low in negative affect (mean = 18.88). We will label it as “COVID negative affect, medium-high gratitude, and good affectivity.”

Kruskal–Wallis *H*-test indicates significant differences (*p* < 0.001) in IG, GS, RG, EG, PA, NA, and FC variables in the second selected cluster analysis. Mann Whitney *U*-test (Table 9) shows that all differences between clusters are significant (*p* < 0.001) for all variables, except between clusters #2 and #3 for the variables IG, GS, RG, EG, PA, and NA (*p* > 0.08).

**Table 9:** Mann-Whitney *U*-test in variables of second selected cluster analysis.

**Clusters**	**IG (*U* | *p*)**	**GS (*U* | *p*)**	**RG (*U* | *p*)**	**EG (*U* | *p*)**	**PA (*U* | *p*)**	**NA (*U* | *p*)**	**FC (*U* | *p*)**
#1–#2	851.5 | 0.000	480 | 0.000	185.5 | 0.000	785 | 0.000	1.009 | 0.000	1.668.5 | 0.000	1.695.5 | 0.000
#1–#3	757 | 0.000	426 | 0.000	202 | 0.000	1.036 | 0.000	1.283.5 | 0.000	1.913 | 0.000	1.741.5 | 0.000
#2–#3	757 | 0.470	426 | 0.771	202 | 0.506	1.036 | 0.171	1.283.5 | 0.090	1.913 | 0.481	1.741.5 | 0.000

The third selected cluster analysis included the variables of COVID affectation and purpose in life variables. The three obtained clusters are described below:

Cluster #1 (*n* = 122, 40.4%) comprises 100% of participants who report being worse since the pandemics started. In purpose in life, they obtained high scores in SSL (mean = 32.88) and in GPL (mean = 22.48); in relation to affect, they obtained high scores in PA (mean = 47.73) and low scores in NA (mean = 17.20). We will label it as “worse in COVID, good purpose in life, and good affectivity.”

Cluster #2 (*n* = 70, 23.2%) comprises 48.6% of participants who report being worse since the pandemic started, the remaining 51.4% are distributed between no change (41.4%) and better (10%). In purpose in life, they obtained medium-low scores in SSL (mean = 21.57) and in GPL (mean = 14.77). In relation to affect, they obtained medium-low scores in positive affect (mean = 33.51) and medium-high scores in negative affect (mean = 29.01). We will label it as “mixed on facing COVID, medium-low purpose in life, and medium affect.”

Cluster #3 (*n* = 110, 36.4%) comprises 87.3% participants reporting no change since the pandemic started, and the remaining 12.7 % are better. In purpose in life, they obtained high scores in SSL (mean = 33.95) and in GPL (mean = 23.23); in relation to affect, they obtained high scores in PA (mean = 49.51) and low scores in NA (mean = 16.38). We will label it as “worse in COVID, good purpose in life, and good affectivity.”

Kruskal–Wallis *H*-test indicates significant differences (*p* < 0.001) in SSL, GPL, PA, NA, and FC variables in the third selected cluster analysis. Mann Whitney *U*-test (Table 10) shows that all differences between clusters are significant (*p* < 0.017) for all variables, except between clusters #1 and #3 for the variables SSL, GPL, and NA (*p* > 0.06).

**Table 10:** Mann-Whitney *U*-test in variables of third selected cluster analysis.

**Clusters**	**PA (*U* | *p*)**	**NA (*U* | *p*)**	**FC (*U* | *p*)**	**SSL (*U* | *p*)**	**GPL (*U* | *p*)**
#1–#2	1,005.5 | 0.000	1,070.5 | 0.000	2,074 | 0.000	519 | 0.000	787.5 | 0.000
#1–#3	5,444.5 | 0.013	6,383.5 | 0.521	0 | 0.000	5,804.5 | 0.075	5,941.5 | 0.131
#2–#3	541 | 0.000	792.5 | 0.000	2,058 | 0.000	287 | 0.000	489 | 0.000

## Discussion and Conclusion

Possible consequences of the COVID-19 pandemic are rather unpredictable; studies conducted during the pandemic allow us to explore the short-term impact of the pandemic and to identify new factors that influence global health. Specifically, we have explored the factors that may influence the subjective perception of the emotional and behavioral impact of COVID-19.

In relation to age, this study found an inverse association between negative affect and age, indicating that the older the age, the lower the negative affect scores. Along the same lines, other studies found older people had better well-being scores than younger people (e.g., Bidzan-Bluma et al., 2020). However, in this study, age did not have significant relationships with positive affect and was not associated with facing COVID-19. In this regard, Ebert et al. (2020), in a study with a comparable sample to our study (participants from crowdsourcing platform, MTurk), found mean age differences were observed, but the trajectory of change did not differ by age. This suggests that responses to COVID-19 may be age-invariant and that effects on well-being are not immediate, but that they may emerge over a longer period of time. For our part, we believe that the most noticeable age-related changes may be in negative affect, as people may learn to manage their emotional distress throughout their life. However, it seems that the activation of positive emotions and the categorization of facing COVID is invariant with age.

Gender also did not show differences in COVID-19 affectation; the three facing COVID-19 groups display similar percentages in men and women. Differences in positive and negative affect have also not been found. In this sense, these results are in addition to those papers in which no differences were found between men and women in terms of COVID-19 affectation (Cao et al., 2020; Huang and Zhao, 2020; Zhang and Ma, 2020) and contrast the outcomes where women are more negatively affected (Rogowska et al., 2020; Tan et al., 2020; Wang et al., 2020). However, the cluster analysis allowed us to find differences between the groups obtained according to gender. Thus, of the five clusters obtained, two clusters (cluster 3 and 4) are similar in all variables, although cluster 3 is composed entirely of women and cluster 4 is composed entirely of men. Cluster 2, on the other hand, is equally mixed in terms of gender, so it does not indicate differences between men and women. Thus, the results of this study could be consistent with the findings above: there are profiles of participants in which they are gender invariant (those who indicated that they did not notice changes since the pandemic started and those who indicated that they were better), and there are other profiles (those who indicated that they were worse) in which there is a gender difference, in the sense that women are more negatively affected. These results have important implications for research because they demonstrate the relevance of classifying participants into profiles, thus further clarifying results when contradictory results were found in previous literature. In sum, more research is needed regarding age and gender as well as controlling labor and childcare variables, since these could be factors affecting well-being in a period of lockdown and crisis.

In relation to personality traits, the results are in line with other COVID-19 studies that show how emotional stability was inversely related to COVID-19 affectation (Aschwanden et al., 2020; Gubler et al., 2020) and with negative affect. Emotionally unstable individuals (i.e., individuals with high levels of neuroticism) have more dysfunctional interpersonal relationships and are less satisfied with their relationships, experience fear, depression, and guilt more often than emotionally stable individuals, and are more sensitive to social rejection cues. Additionally, higher conscientiousness was associated negatively with COVID-19 affectation. This result aligns with previous research that found individuals with a high conscientiousness took more precautions to avoid catching coronavirus (Aschwanden et al., 2020). This circumstance may have influenced the impact of the pandemic, since new behavioral habits aimed at preventing infection have been acquired, which has influenced their adaptation. Thus, the research of personality traits and coping with the COVID-19 pandemic is an emerging research area that can help advise public healthcare policy recommendations, taking into account personality traits and their response to healthcare.

On the other hand, gratitude, purpose in life, and religiosity did not obtain associations with facing COVID-19. However, these three constructs were significantly related to positive and negative affect experienced during the pandemic.

Specifically, the results reveal that all four subscales of gratitude were positively associated with positive affect and inversely associated with negative affect, indicating that people who are more grateful, both to other people and to transcendental forces, experience a better affective experience. This result is consistent with previous studies in which gratitude was related to various dimensions of well-being, conducted throughout the COVID-19 pandemic (e.g., Burke et al., 2020; Jiang, 2020), in other historical times of adversity (e.g., Coffman, 1996; Peterson and Seligman, 2003), and also in normative historical times (e.g., Mairean et al., 2019). However, the four subscales of gratitude were not related to FC. We suggest that this result could be due to the specificity of the measure, which asks specifically about the experience of COVID-19 and not about the general state of personal well-being. Along the same lines, we found the results of a study in which a scale was designed specifically for the COVID-19 situation. It included nine gratitude items and the participants were asked, “In the past month, how much has your experience of the COVID-19 crisis led you to feel grateful for the following things?” The results did not reveal significant associations between their COVID-19 specific gratitude scale and scores on depression, anxiety, and stress, but they did find significant associations with personal well-being and with their perception of COVID-19 (Burke et al., 2020). These results show us the importance of taking great care in interpreting the results in terms of the specificity of the measure, in order to be able to differentiate accurately whether gratitude is measured at the trait level or whether gratitude is measured for specific situations, as different patterns of associations may emerge for different specifications of gratitude. In any case, these results show the importance in affectivity of the two types of gratitude measured (interpersonal and transcendental), and the different processes it involves (gratitude in the face of suffering, re-conception of gifts and expression of gratitude). Of particular note is the novel facet of gratitude used in this study on gratitude in the face of suffering, which allows us to value gratitude in spite of adversity. This facet has even obtained higher correlations with PA and NA than interpersonal gratitude, a facet that is usually associated to a greater extent with well-being, because it affects interpersonal relationships. It may be that in times of adversity, such as a global pandemic, this facet of gratitude could play an important role in maintaining a good affective experience.

In addition, gratitude was one of the constructs studied that allowed us to classify the participants into profiles according to their COVID affect. Thus, the three clusters resulting from the analysis show that cluster 2, labeled as “no COVID affect, medium-high gratitude, and good affect” and cluster 3, labeled as “negative COVID affect, medium-high gratitude, and good affect” indicate good indices of gratitude and good affect (characterized by high scores on positive affect and low scores on negative affect). That is, the only difference was that participants in cluster 3 responded that they were “worse” in relation to the pandemic, and those in cluster 2 experienced “no change” or were “better,” but the scores in both groups on Positive Affect (PA) and Negative Affect (NA) and gratitude were similar, indicating good gratitude and good affectivity. There are several possible explanations for this: it could be that cluster 3 participants had higher previous levels of affectivity and that, in the face of the COVID-19, their affective experience worsened, now equating to the affectivity of cluster 2 participants, whose previous levels of affectivity could be similar to those obtained during the pandemic, this being congruent with their “no change” response. In other words, from this explanation, very high basal levels of affectivity might decrease in the face of the pandemic and match high levels, while high basal levels might be maintained. Another possible explanation could be related to response biases, specifically related to the global-specificity of cognitive judgements; some people might be more congruent between their global judgements about their emotional-affective experience in relation to judgements of affect and concrete experiences (measured, for example, with the PANAS and G20 questionnaires), while others might opt for a global judgement that overestimates or underestimates their concrete affective experience, thus not corresponding to both measures. Research has studied this relationship and has found that specific judgments were slightly more accurate than global judgments (Karst et al., 2018).

In this sense, cluster 1, labeled as “mixed in facing COVID, medium gratitude, and medium affectivity” does differ from the other clusters with lower values for gratitude and affectivity (less positive affect and more negative affect). It is worth noting that cluster 1 is made up of a mixture of participants who responded that they were “worse,” “no change,” and “better” in relation to the pandemic. Thus, in the group of participants with worse affect, 14% of them indicated that they felt better over the period of the pandemic, which could be an overestimation as a strategy to enhance well-being and resilience. This optimistic view may represent an adaptive “distortion” of reality that fosters people's mental health (Colombo et al., 2020). Thirty-two percent of participants indicated that they had not noticed changes in the pandemic, although they scored medium on gratitude and PA and higher on NA than the other two clusters. Regarding this connection, a previous study showed that the presence of mild depressive symptoms led participants to a greater overestimation of NA and higher underestimation of PA (Colombo et al., 2019). This could be the case for this group of participants, whose affectivity is worse and who cannot enjoy the potential benefits of gratitude. In any case, and despite the biases, what is clear is that participants can be grouped into two distinct profiles in relation to gratitude: those with good affect who have high scores on gratitude, and those with medium-low scores on positive affect and medium-high scores on negative affect with medium gratitude. These results could be interpreted because grateful people value acts of altruism, help, and sacrifice that have been experienced during the COVID-19 pandemic and may reinforce belief in positive human nature, leading to a better affective experience. Thus, gratitude emerges as a strength that can be promoted through interventions and thus increase, for example, happiness (Dickens, 2017), well-being (e.g., Wood et al., 2010; Rash et al., 2011), and physical health (Boggiss et al., 2020). For example, during the pandemic, the Department of Surgery at the University of California, San Francisco (UCSF) implemented the “Gratitude and Good Outcomes” program with the purpose of allowing Department members to publicly express gratitude and to highlight and celebrate examples of outstanding teamwork and surgical skill, as demonstrated by our surgeons and their teams. This demonstrates the effectiveness of taking some time to recognize and celebrate good work, which should be an essential component of clinician training and practice, thus providing care tools for such an important professional sector in times of a pandemic. Additionally, in the field of education, evidence has been found that programs aimed at increasing trait gratitude in adolescents improved well-being for 6 weeks after said interventions (Bono et al., 2020b). Thus, the development of gratitude is configured as a strategy with very important practical implications in various contexts, such as health and education.

In relation to purpose in life, this was not related to FC, but it did have significant positive associations with PA. Similarly, studies such as that by Trzebiński et al. (2020) found that high meaning in life and life satisfaction, as well as strong presumptions on orderliness and positivity of the world, correlate with fewer panic thoughts and emotions evoked by the apparent danger of the ongoing COVID-19 pandemic. On the other hand, NA was also positively and significantly associated with the two subscales of PIL. Previous studies have found that the purpose of life has been related to greater well-being (e.g., Işik and Üzbe, 2015), more positive affect, and less negative affect. However, it is suggested that psychological interventions aimed at re-signifying experiences and finding new meaning in their lives could help to cushion the negative effects of the pandemic situation. For example, it has been proposed that life crafting interventions could offer a way to help people cope and renew their sense of life (De Jong et al., 2020). In relation to the cluster analysis between purpose in life and COVID affectation, three clusters are obtained with the same structure as the clusters obtained with the gratitude variables, in which the group with the worst affectivity and meaning of life, categorizes—in a biased way—COVID affectation, while another group that indicates that it feels worse since the pandemic, obtained good affectivity. We assume the same aforementioned argument in relation to the influence of affect on response biases.

With regard to religiosity and COVID affectation, the results show that there is no relationship in terms of FC, but significant associations were obtained with PA in a positive way and with inverse NP. These results are in line with previous research, such as that by Lucchetti et al. (2020) who found—in a sample from Brazil—that lower levels of worrying in the pandemic were associated with greater private religious activities, religious attendance, spiritual growth, and with an increase in religious activities; lower levels of fear were associated with greater private religious activities and spiritual growth. Lower levels of sadness were associated with spiritual growth. Another study also found that the well-being of tumor patients during the pandemic was predicted by a mix of disease and pandemic related stressors, and by available resources such as meaning in life and religious trust (Büssing et al., 2020). Thus, religiosity could be a buffer for stressors in the pandemic, although more research is needed, for example, on specific confessionality and a cross-cultural approach to further delineate these relationships.

Moreover, this study found significant associations between gratitude, purpose in life, and religiosity, similar to other studies conducted in non-pandemic times (e.g., Bernabé Valero, 2012). These results suggest that the underlying patterns of human strength relationships are maintained despite adverse situations, such as COVID-19. In this way, psychologists and researchers could take these relational patterns into account when designing interventions aimed at enhancing each strength and improving the affective experience.

In summary, the results of this paper have important implications for research, which have been developed throughout this discussion, as they alert us to potential biases in the measurement of affectivity and encourage future work to consider baseline levels of affect and to use multi-method and multi-source strategies to control for such biases. Similarly, another limitation of this study is the cross-sectional design that does not allow for the assessment of changes over time. Nevertheless, our results corroborate the importance of further research on the identification of individual differences to guide public health policy decisions and the actions of physical and mental health professionals.

## Data Availability Statement

The original contributions presented in the study are included in the article/Supplementary Materials, further inquiries can be directed to the corresponding author.

## Ethics Statement

The studies involving human participants were reviewed and approved by Universidad Católica de Valencia San Vicente Mártir committee (number UCV2017-2018-28). The patients/participants provided their written informed consent to participate in this study.

## Author Contributions

GB-V conceived the presented idea. GB-V, DM-F, ID, and MG developed the theory and performed the computations. All authors discussed the results and contributed to the final manuscript.

## Conflict of Interest

The authors declare that the research was conducted in the absence of any commercial or financial relationships that could be construed as a potential conflict of interest.

## References

[B1] Ammar A. Chtourou H. Boukhris O. Trabelsi K. Masmoudi L. Brach M.. (2020). COVID-19 home confinement negatively impacts social participation and life satisfaction: a worldwide multicenter study. Int. J. Environ. Res. Public Health 17:6237. 10.3390/ijerph17176237 PMC7503681 32867287

[B2] Arslan G. Yildirim M. Wong P. T. P. (2020). Meaningful living, resilience, affective balance, and psychological health problems during COVID-19. PsyArXiv. 10.31234/osf.io/wsr3e PMC7785475 33424205

[B3] Aschwanden D. Strickhouser J. E. Sesker A. A. Lee J. H. Luchetti M. Stephan Y.. (2020). Psychological and behavioural responses to coronavirus disease 2019: the role of personality. Eur. J. Personal. 10.1002/per.2281. [Epub ahead of print]. 32836766 PMC7361622

[B4] Bentzen J. (2020). In Crisis, We Pray: Religiosity and the COVID-19 Pandemic. Available online at: https://www.researchgate.net/publication/343627578_In_Crisis_We_Pray_Religiosity_and_the_COVID-19_Pandemic 10.1016/j.jebo.2021.10.014 PMC8557987 34744223

[B5] Bernabé Valero M. G. (2012). La gratitud como actitud existencial: papel predictivo de la religiosidad, la espiritualidad y el sentido de la vida (Tesis Doctoral). Universidad Católica de Valencia San Vicente Mártir, Valencia.

[B6] Bernabe-Valero G. Blasco-Magraner J. S. García-March M. R. (2020). Gratitude questionnaire-20 items (G20): a cross-cultural, psychometric and crowdsourcing analysis. Front. Psychol. 11:626330. 10.3389/fpsyg.2020.626330 33408677 PMC7779484

[B7] Bernabé-Valero G. García-Alandete J. Gallego-Pérez J. F. (2014). Construcción de un cuestionario para la evaluación de la gratitud: el Cuestionario de Gratitud−20 ítems (G-20). Ann. Psychol. 30, 278–286. 10.6018/analesps.30.1.135511

[B8] Bernabé-Valero G. Moret-Tatay C. García-Alandete J. (2015). Análisis confirmatorio de una escala para la medida de la religiosidad: la Escala Breve de Religiosidad (EBR). Psiquiatría. com. 1:28. Available online at: https://psiquiatria.com/bibliopsiquis/volumen.php?wurl=analisis-confirmatorio-de-una-escala-para-la-medida-de-la-religiosidad-la-escala-breve-de-religiosidad-ebr

[B9] Bidzan-Bluma I. Bidzan M. Jurek P. Bidzan L. Knietzsch J. Stueck M.. (2020). A polish and German population study of quality of life, well-being, and life satisfaction in older adults during the COVID-19 pandemic. Front. Psychiatry 11:585813. 10.3389/fpsyt.2020.585813 33281646 PMC7705096

[B10] Boggiss A. L. Consedine N. S. Brenton-Peters J. M. Hofman P. L. Serlachius A. S. (2020). A systematic review of gratitude interventions: effects on physical health and health behaviors. J. Psychosom. Res. 135:110165. 10.1016/j.jpsychores.2020.110165 32590219

[B11] Bono G. Emmons R. A. McCullough M. E. (2004). “Gratitude in practice and the practice of gratitude,” in Positive Psychology in Practice, eds P. A. Linley and S. Joseph (John Wiley & Sons, Inc), 464–481.

[B12] Bono G. Mangan S. Fauteux M. Sender J. (2020a). A new approach to gratitude interventions in high schools that supports student wellbeing. J. Posit. Psychol. 15, 657–665. 10.1080/17439760.2020.1789712

[B13] Bono G. Reil K. Hescox J. (2020b). Stress and wellbeing in college students during the COVID-19 pandemic: can grit and gratitude help? Int. J. Wellbeing 10, 39–57. 10.5502/ijw.v10i3.1331

[B14] Burke T. Berry A. Taylor L. K. Stafford O. Murphy E. Shevlin M.. (2020). Increased psychological distress during COVID-19 and quarantine in Ireland: a national survey. J. Clin. Med. 9:3481. 10.3390/jcm9113481 33126707 PMC7693396

[B15] Büssing A. Hübner J. Walter S. Gießler W. Büntzel J. (2020). Tumor patients' perceived changes of specific attitudes, perceptions, and behaviors due to the COVID-19 pandemic and its relation to reduced wellbeing. Front. Psychiatry 11:574314. 10.3389/fpsyt.2020.574314 33192703 PMC7581913

[B16] Cao W, Fang, Z, Hou, G, Han, M, Xu, X, Dong, J. (2020). The psychological impact of the COVID-19 epidemic on college students in China. Psychiatry Res. 287:112934. 10.1016/j.psychres.2020.112934 32229390 PMC7102633

[B17] Coffman S. (1996). Parents' struggles to rebuild family life after hurricane Andrew. Issues Ment. Health Nurs. 17, 353–367. 10.3109/01612849609009406 8920336

[B18] Colombo D. Suso-Ribera C. Fernández-Álvarez J. Cipresso P. Garcia-Palacios A. Riva G.. (2020). Affect recall bias: being resilient by distorting reality. Cognit. Ther. Res. 44, 906–918. 10.1007/s10608-020-10122-3

[B19] Colombo D. Suso-Ribera C. Fernandez-Álvarez J. Felipe I. F. Cipresso P. Palacios A. G.. (2019). “Exploring affect recall bias and the impact of mild depressive symptoms: an ecological momentary study,” in Pervasive Computing Paradigms for Mental Health, eds P. Cipresso, S. Serino, and D. Villani (New York, NY: Springer International Publishing), 208–215.

[B20] Crumbaugh J. C. (1968). Cross-validation of purpose-in-life test based on Frankl's concepts. J. Individ. Psychol. 24:74. 10.1037/t01175-000 4385494

[B21] De Jong E. M. Ziegler N. Schippers M. C. (2020). From shattered goals to meaning in life: Life crafting in times of the COVID-19 pandemic. Front. Psychol. 11:2648. 10.3389/fpsyg.2020.577708 33178081 PMC7593511

[B22] Dickens L. R. (2017). Using gratitude to promote positive change: a series of meta-analyses investigating the effectiveness of gratitude interventions. Basic Appl. Soc. Psych. 39, 193–208. 10.1080/01973533.2017.1323638

[B23] Ebert A. R. Bernstein L. E. Carney A. K. Patrick J. H. (2020). Emotional well-being during the first four months of COVID-19 in the United States. J. Adult Dev. 27, 241–248. 10.1007/s10804-020-09365-x 33132677 PMC7584310

[B24] Fitzgerald P. (1998). Gratitude and justice. Ethics 109, 119–153. 10.1086/233876

[B25] Frankl V. E. (1985). Man's Search for Meaning. New York, NY: Simon and Schuster.

[B26] Fredrickson B. L. Tugade M. M. Waugh C. E. Larkin G. R. (2003). What good are positive emotions in crisis? A prospective study of resilience and emotions following the terrorist attacks on the United States on September 11th, 2001. J. Pers. Soc. Psychol. 84, 365–376. 10.1037/0022-3514.84.2.365 12585810 PMC2755263

[B27] García-Alandete J. Martínez E. R. Soucase Lozano B. Gallego-Pérez J. F. (2011). Diferencias asociadas al sexo en las puntuaciones total y factoriales del Purpose-In-Life Test en universitarios españoles. Universitas Psychol. 10, 681–692. 10.11144/Javeriana.upsy10-3.dasp

[B28] Gosling S. D. Rentfrow P. J. Swann W. B. Jr. (2003). A very brief measure of the Big-Five personality domains. J. Res. Pers. 37, 504–528. 10.1016/S0092-6566(03)00046-128810502

[B29] Gubler D. A. Makowski L. M. Troche S. J. Schlegel K. (2020). Loneliness and well-being during the Covid-19 pandemic: associations with personality and emotion regulation. J. Happiness Stud. 10.1007/s10902-020-00326-5. [Epub ahead of print]. 33100896 PMC7574670

[B30] Harlow L. L. Newcomb M. D. Bentler P. M. (1987). Purpose in Life Test assessment using latent variable methods. Br. J. Clin. Psychol. 26, 235–236. 10.1111/j.2044-8260.1987.tb01355.x 3664045

[B31] Huang Y. Zhao N. (2020). Generalized anxiety disorder, depressive symptoms and sleep quality during COVID-19 outbreak in China: a web-based cross-sectional survey. Psychiatry Res. 288:112954. 10.1016/j.psychres.2020.112954 32325383 PMC7152913

[B32] Işik S. Üzbe N. (2015). Personality traits and positive/negative affects: an analysis of meaning in life among adults. Educ. Sci. Theory Prac. 15, 587–595. 10.12738/estp.2015.3.2436

[B33] Jiang D. (2020). Feeling gratitude is associated with better well-being across the life span: a daily diary study during the COVID-19 outbreak. J. Gerontol. Ser. B. 10.1093/geronb/gbaa220. [Epub ahead of print]. PMC7798492 33284976

[B34] Karst K. Dotzel S. Dickhäuser O. (2018). Comparing global judgments and specific judgments of teachers about students' knowledge: Is the whole the sum of its parts? Teach. Teach. Educ. 76, 194–203. 10.1016/j.tate.2018.01.013

[B35] Kashdan T. B. Uswatte G. Julian T. (2006). Gratitude and hedonic and eudaimonic well-being in Vietnam war veterans. Behav. Res. Ther. 44, 177–199. 10.1016/j.brat.2005.01.005 16389060

[B36] Kaufman L. Rousseeuw P. J. (Eds.). (1990). Finding Groups in Data: An Introduction to Cluster Analysis. New York, NY: John Wiley and Sons.

[B37] Kowal M. Coll-Martin T. Ikizer G. Rasmussen J. Eichel K. Studzinska A.. (2020). Who is the most stressed during the COVID-19 pandemic? Data from 26 countries and areas. Appl. Psychol. Health Well Being 12, 946–966. 10.1111/aphw.12234 32996217 PMC7537225

[B38] Kranz D. Niepel C. Botes E. Greiff S. (2020). Religiosity predicts unreasonable coping with COVID-19. Psychol. Religion Spiritual. 10.1037/rel0000395

[B39] Lucchetti G. Góes L. G. Amaral S. G. Ganadjian G. T. Andrade I. Almeida P.. (2020). Spirituality, religiosity and the mental health consequences of social isolation during Covid-19 pandemic. Int. J. Soc. Psychiatry. 10.1177/0020764020970996. [Epub ahead of print]. 33135559 PMC7649649

[B40] Mairean C. Turliuc M. N. Arghire D. (2019). The relationship between trait gratitude and psychological wellbeing in university students: the mediating role of affective state and the moderating role of state gratitude. J. Happiness Stud. 20, 1359–1377. 10.1007/s10902-018-9998-7

[B41] Martínez-Martí M. L. Theirs C. I. Pascual D. Corradi G. (2020). Character strengths predict an increase in mental health and subjective well-being over a one-month period during the COVID-19 pandemic lockdown. Front. Psychol. 11:584567. 10.3389/fpsyg.2020.584567 33192913 PMC7609545

[B42] Modersitzki N. Phan L. Kuper N. Rauthmann J. F. (2020). Who is impacted? Personality predicts individual differences in psychological consequences of the COVID-19 pandemic in Germany. Soc. Psychol. Personal. Sci. 10.31234/osf.io/s65ux

[B43] Nowicki G. J. Slusarska B. Tucholska K. Naylor K. Chrzan-Rodak A. Niedorys B. (2020). The severity of traumatic stress associated with COVID-19 pandemic, perception of support, sense of security, and sense of meaning in life among nurses: research protocol and preliminary results from Poland. Int. J. Environ. Res. Public Health 17:6491. 10.3390/ijerph17186491 32906590 PMC7559728

[B44] Peterson C. Seligman M. E. (2003). Character strengths before and after September 11. Psychol. Sci. 14, 381–384. 10.1111/1467-9280.24482 12807415

[B45] Rash J. A. Matsuba M. K. Prkachin K. M. (2011). Gratitude and well-being: who benefits the most from a gratitude intervention? Appl. Psychol. Health Well Being 3, 350–369. 10.1111/j.1758-0854.2011.01058.x

[B46] Rogowska A. M. Kuśnierz C. Bokszczanin A. (2020). Examining anxiety, life satisfaction, general health, stress and coping styles during COVID-19 pandemic in Polish sample of university students. Psychol. Res. Behav. Manag. 13, 797–811. 10.2147/PRBM.S266511 33061695 PMC7532061

[B47] Sun N. Wei L. Shi S. Jiao D. Song R. Ma L.. (2020). A qualitative study on the psychological experience of caregivers of COVID-19 patients. Am. J. Infect. Control. 48, 592–598. 10.1016/j.ajic.2020.03.018 32334904 PMC7141468

[B48] Tan Y. Lin X. Wu D. Chen H. Jiang Y. He T.. (2020). Different trajectories of panic and the associated factors among unmarried Chinese during the COVID-19 pandemic. Appl. Psychol. Health Well Being 12, 967–982. 10.1111/aphw.12238 33016617 PMC7675528

[B49] Trzebiński J. Cabański M. Czarnecka J. Z. (2020). Reaction to the COVID-19 pandemic: the influence of meaning in life, life satisfaction, and assumptions on world orderliness and positivity. J. Loss Trauma 25, 544–557. 10.1080/15325024.2020.1765098

[B50] Wang C. Pan R. Wan X. Tan Y. Xu L. Ho C. S.. (2020). Immediate psychological responses and associated factors during the initial stage of the 2019 coronavirus disease (COVID-19) epidemic among the general population in China. Int. J. Environ. Res. Public Health 17:1729. 10.3390/ijerph17051729 32155789 PMC7084952

[B51] Watson D. Clark L. A. (1999). The PANAS-X: Manual for the positive and negative affect schedule-expanded form.

[B52] Wood A. M. Froh J. J. Geraghty A. W. (2010). Gratitude and well-being: a review and theoretical integration. Clin. Psychol. Rev. 30, 890–905. 10.1016/j.cpr.2010.03.005 20451313

[B53] Zhang Y. Ma Z. F. (2020). Impact of the COVID-19 pandemic on mental health and quality of life among local residents in Liaoning Province, China: a cross-sectional study. Int. J. Environ. Res. Public Health 17:2381. 10.3390/ijerph17072381 32244498 PMC7177660

[B54] Zheng L. Miao M. Gan Y. (2020). Perceived control buffers the effects of the COVID-19 pandemic on general health and life satisfaction: the mediating role of psychological distance. Appl. Psychol. Health Well Being 12, 1095–1114. 10.1111/aphw.12232 32955170 PMC7537495

