# Supplementary figures and images for: Individual Differences Facing the COVID-19 Pandemic: The Role of Age, Gender, Personality, and Positive Psychology
Source: Front Psychol. 2021 Mar 19;12:644286. doi: 10.3389/fpsyg.2021.644286 (PMC8012731; doi:10.3389/fpsyg.2021.644286)

**CLUSTER #1**


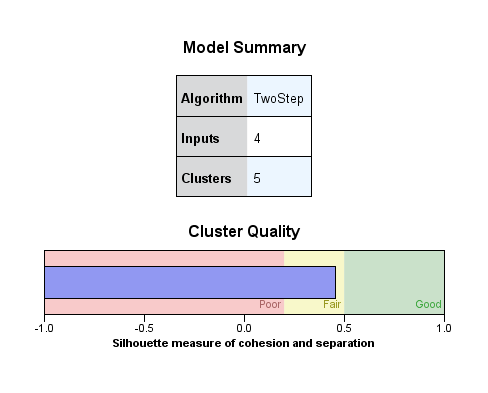


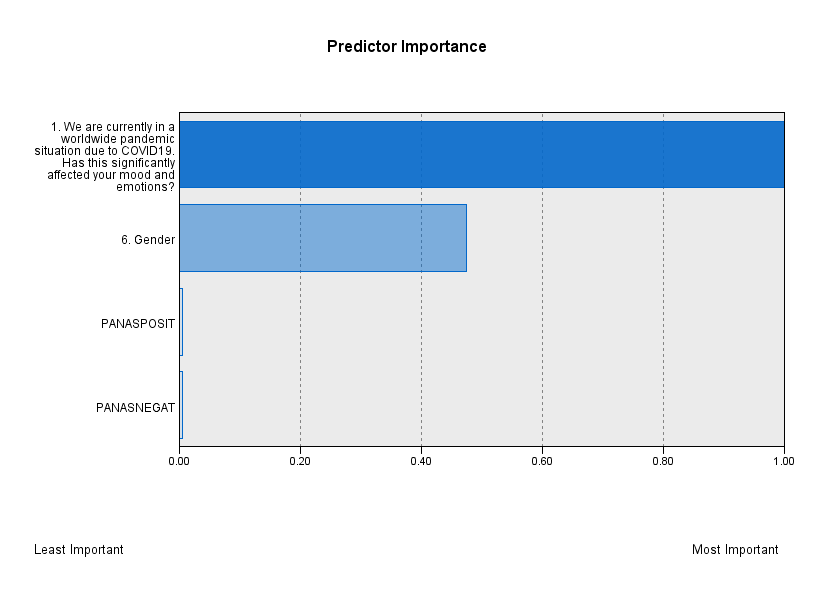


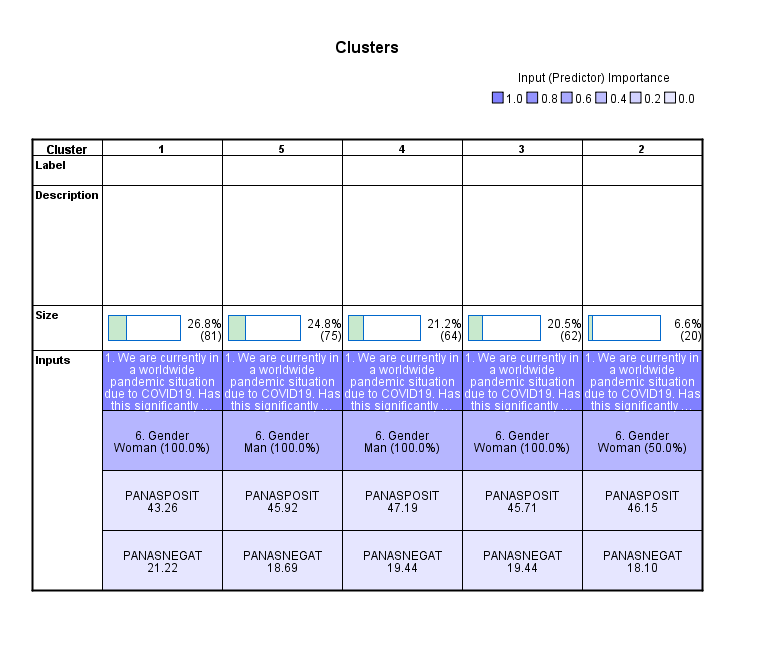


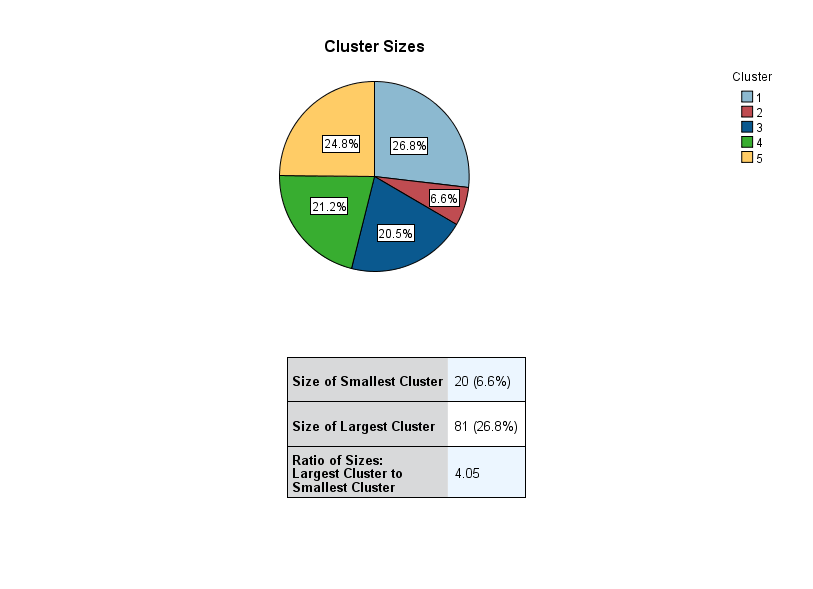


**CLUSTER #2**

**
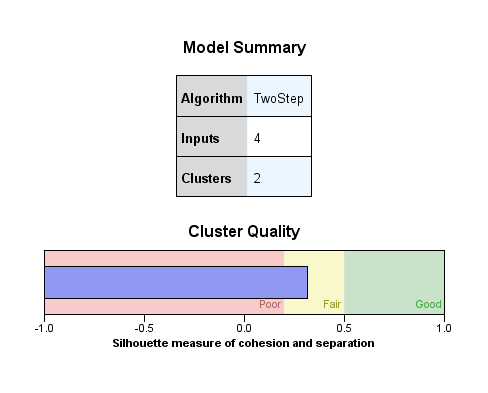
**

**
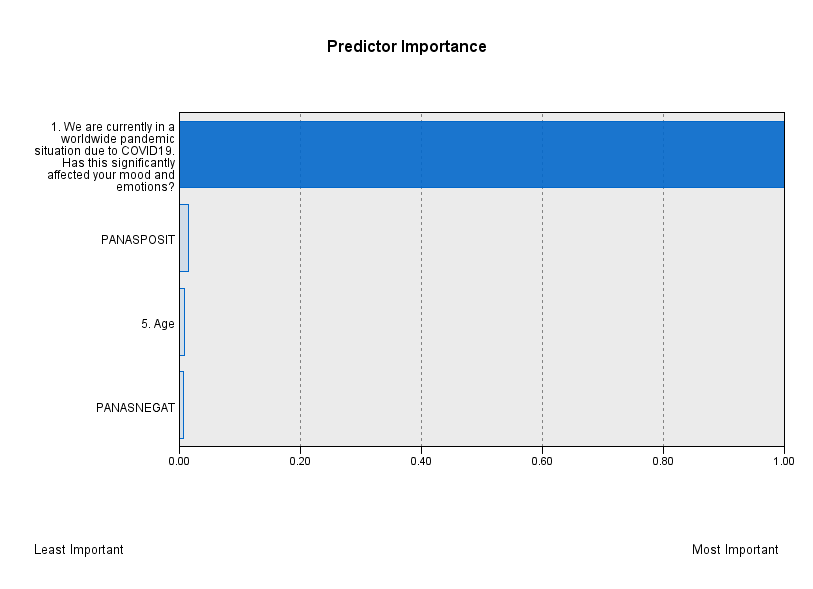
**

**
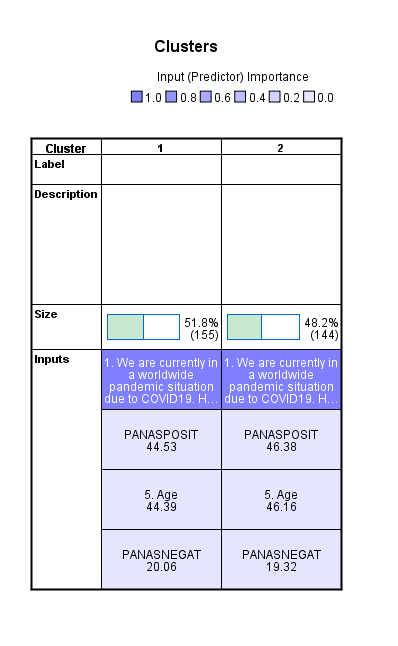

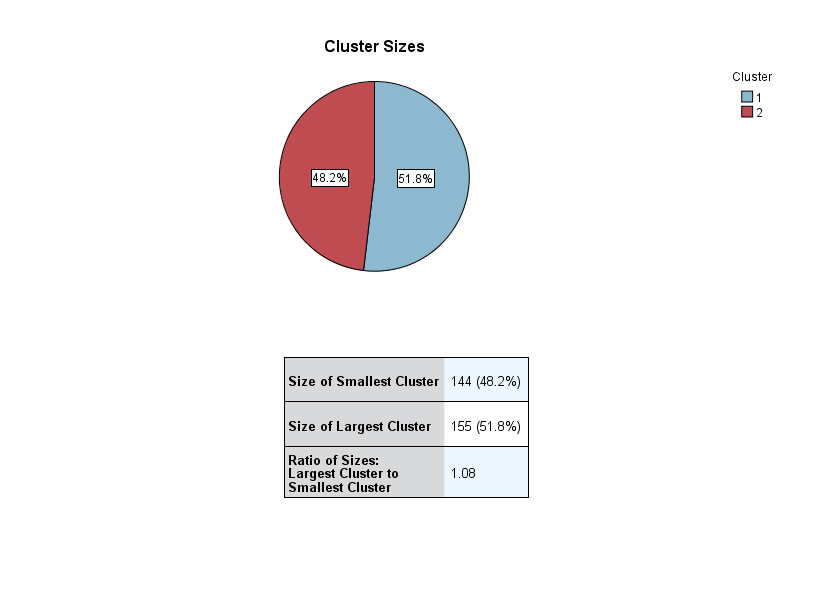
**

**CLUSTER #3**


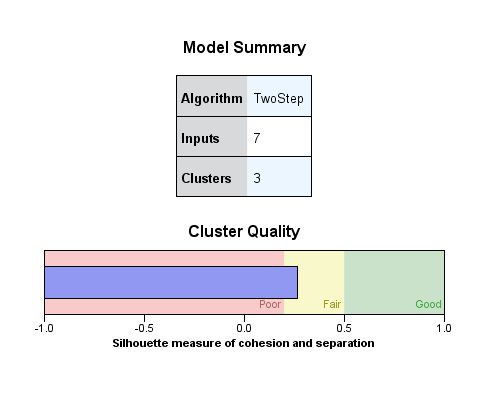


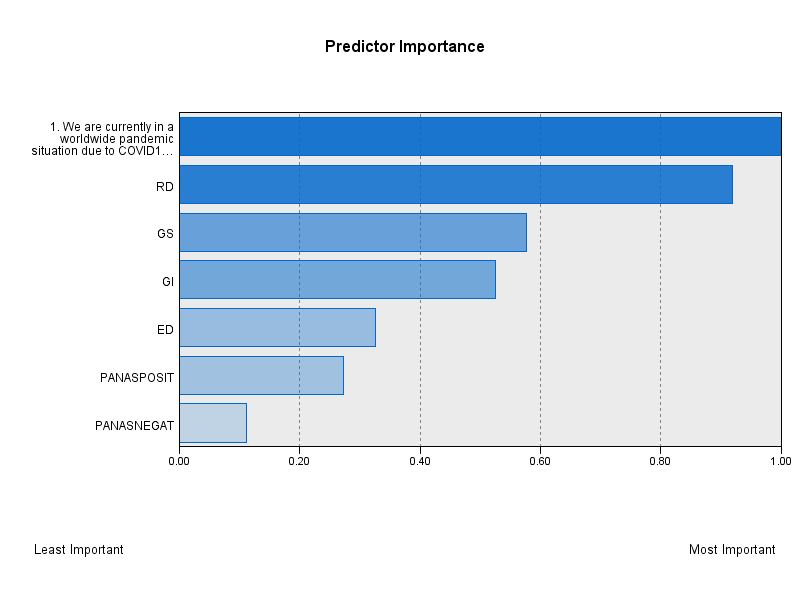


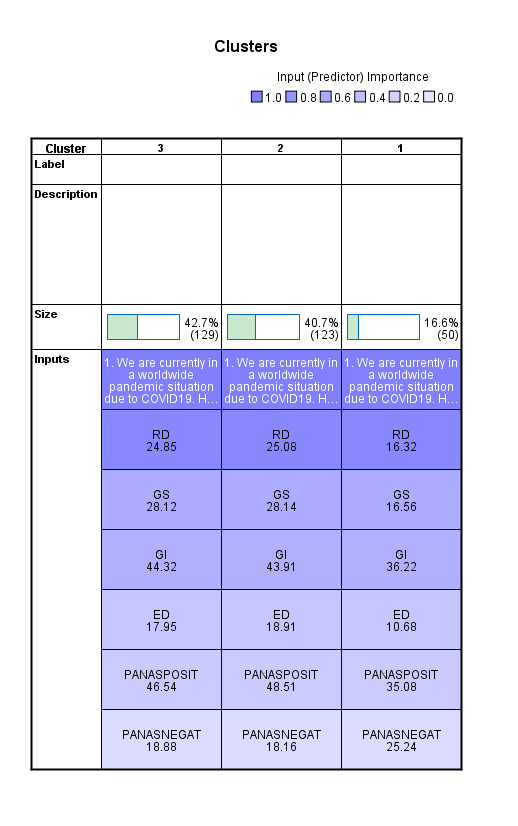


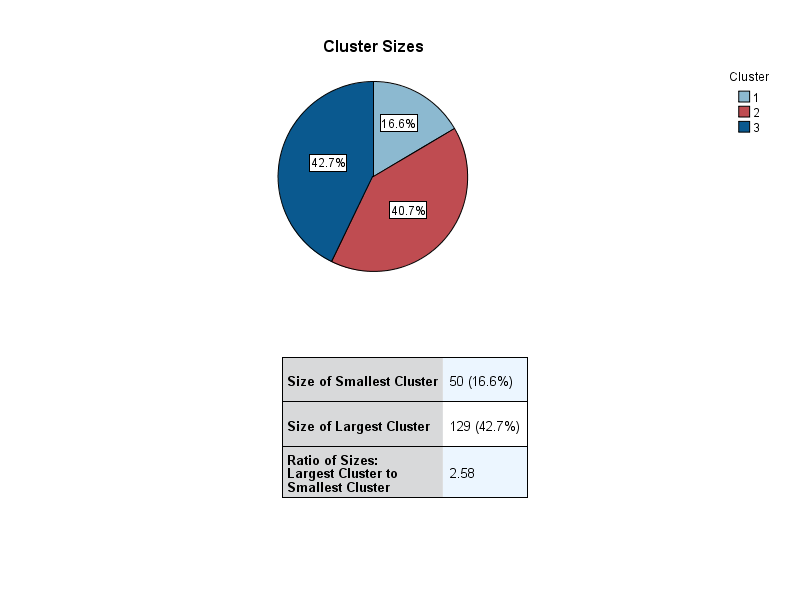


**CLUSTER #4**

**
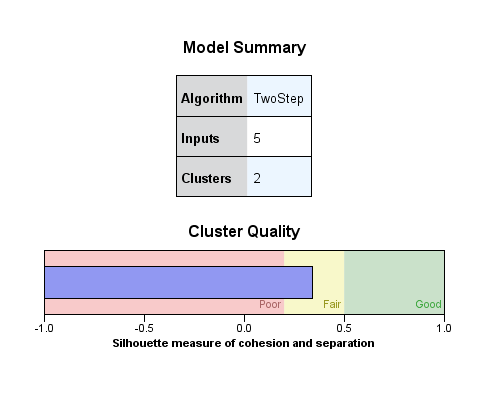
**

**
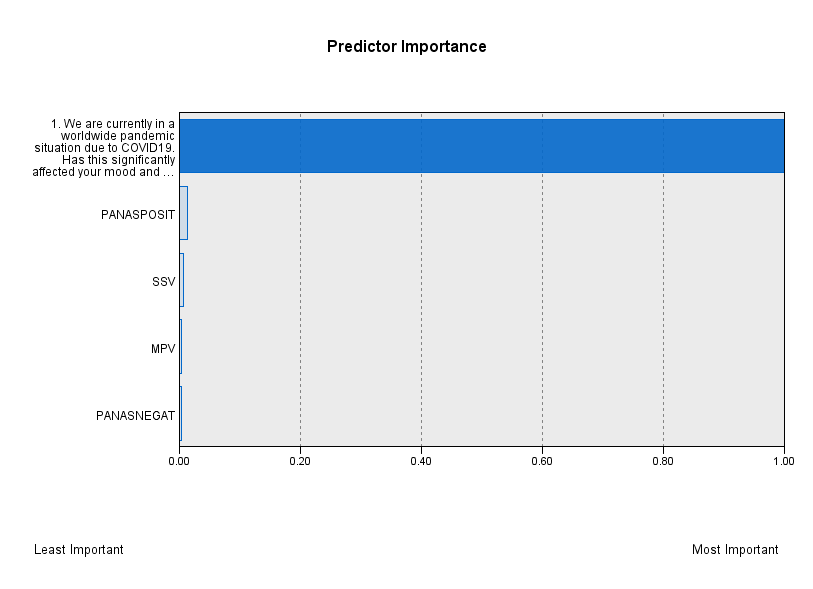
**

**
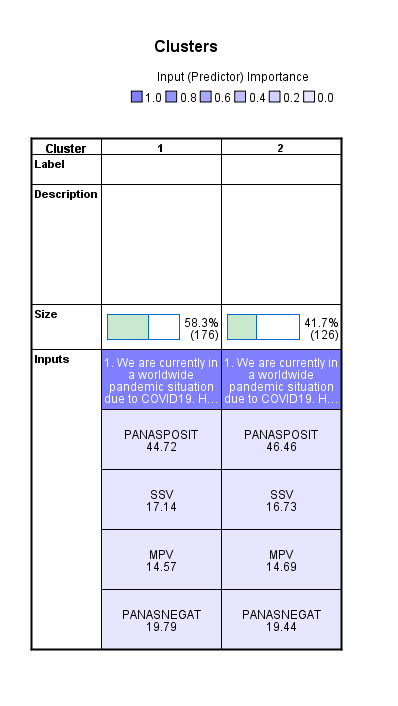
** **
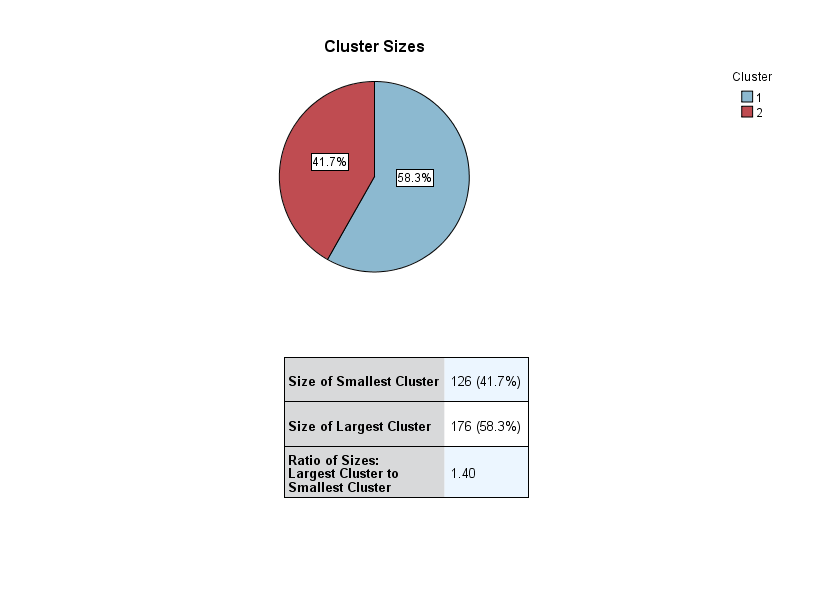
CLUSTER #5**


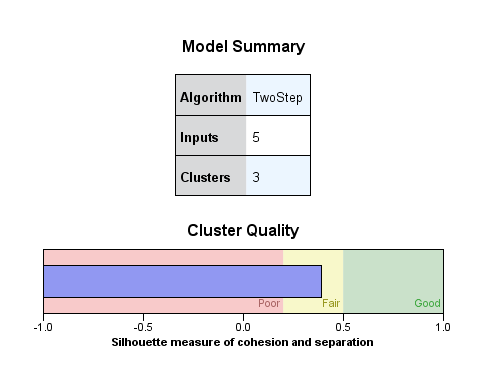


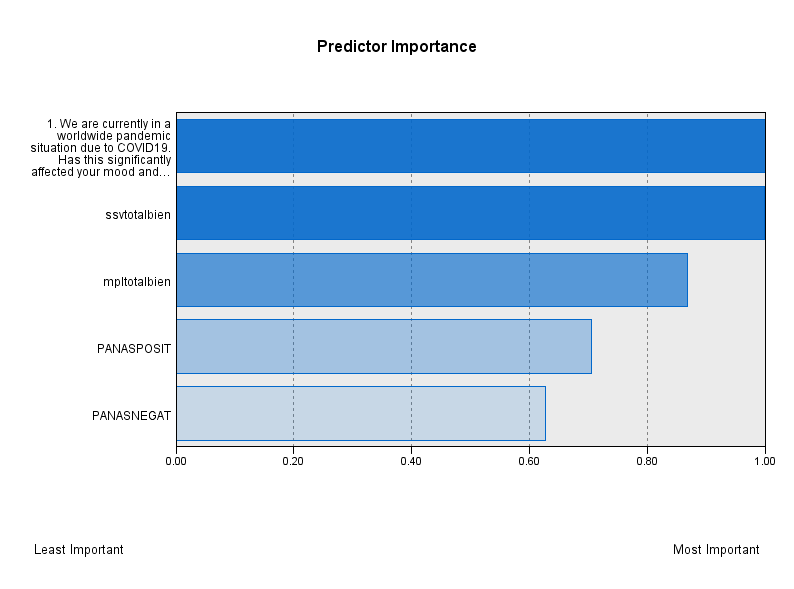


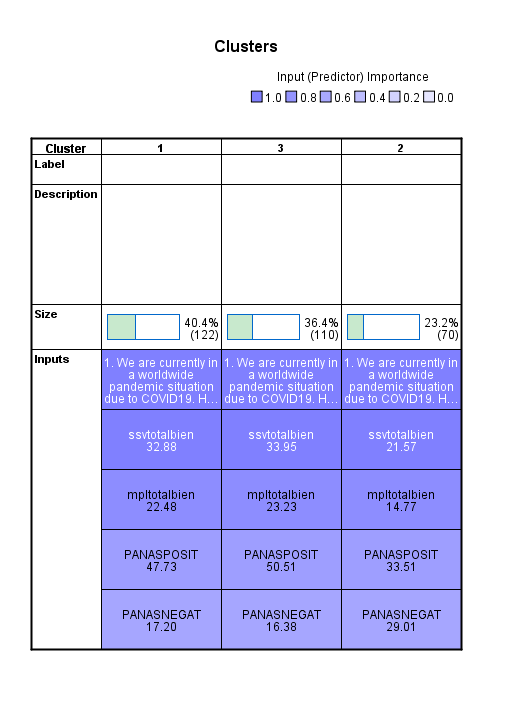


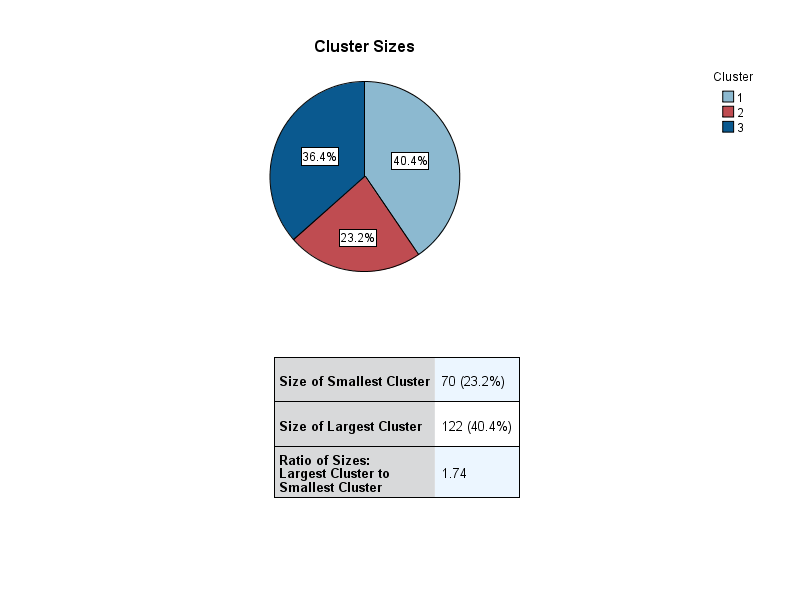


**CLUSTER #6**


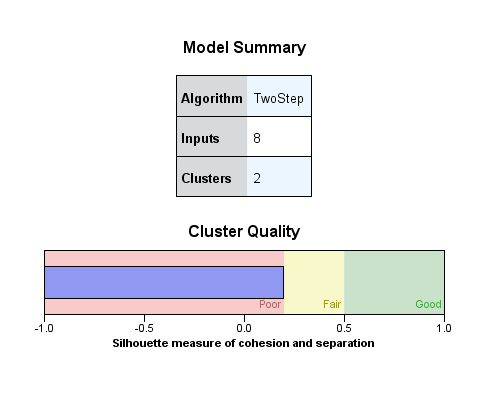


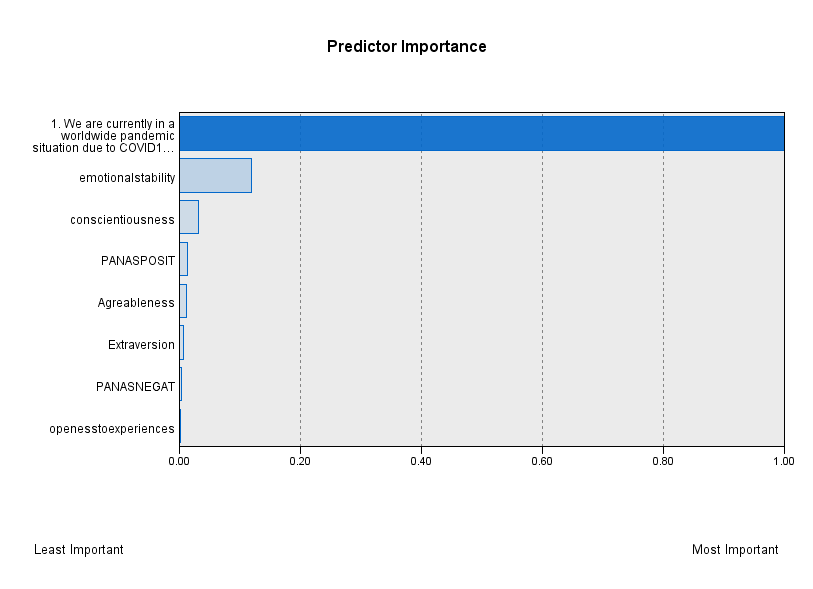


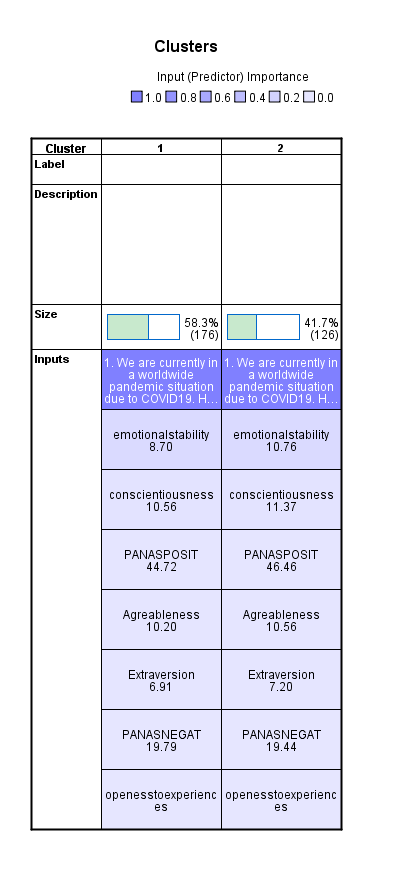

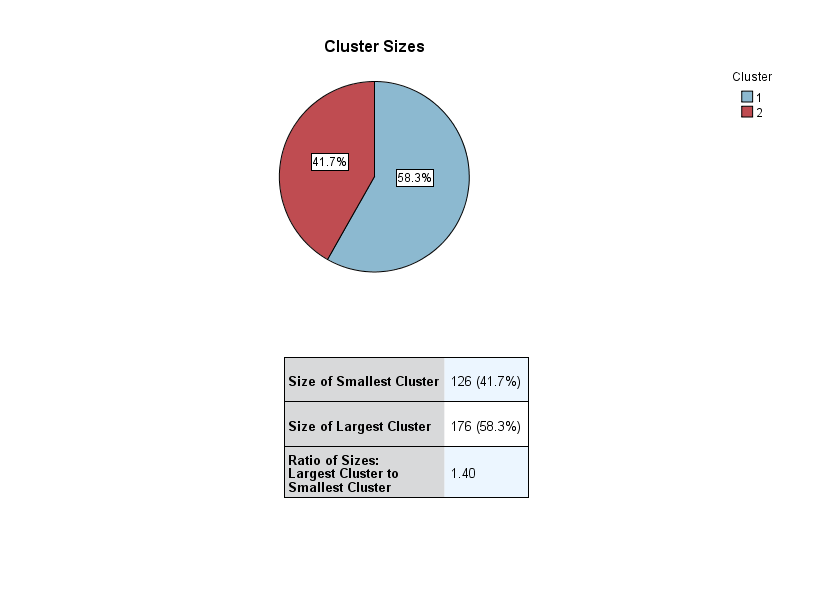

Supplement: Supplementary file 1 [file Data_Sheet_1.docx]
